# Exposures associated with tuberculosis presentation and healthcare delays in South West England, 2015–2020

**DOI:** 10.1371/journal.pone.0340187

**Published:** 2026-01-07

**Authors:** Gemma Lyness, Neville Q. Verlander, Charles R. Beck

**Affiliations:** 1 UK Field Epidemiology Training Programme, UK Health Security Agency (UKHSA), London, United Kingdom; 2 Field Service South West, UK Health Security Agency (UKHSA), London, United Kingdom; 3 Evaluation and Epidemiological Science, UK Health Security Agency (UKHSA), London, United Kingdom; 4 National Institute for Health Research Health Protection Research Unit in Evaluation and Behavioural Science, University of Bristol, Bristol, United Kingdom; 5 Statistics Unit, UK Health Security Agency (UKHSA), London, United Kingdom; Bilawal Medical College, Liaquat University of Medical and Health Sciences, PAKISTAN

## Abstract

Significant progress has been made in reducing the tuberculosis (TB) rate in England over the last decade. South West England has a low incidence of TB, but over a third of people with pulmonary TB (pTB) had a treatment delay of over four months in 2020, higher than the England average. This study aimed to identify the exposures associated with presentation and healthcare delays in receiving pTB treatment in the South West of England between 2015 and 2020. This retrospective cohort study included all confirmed persons with TB resident in South West England, receiving treatment between 2015 and 2020. Univariate and multivariable Cox proportional hazards ratios were produced for the outcome measures of presentation and healthcare delays for persons with pTB. Multivariable regression models were fitted using a forward, stepwise procedure. Sensitivity analyses excluded treatment delays over two years and the year 2020. Data were analysed using Stata 17. Between 2015 and 2020 there were 812 persons with pTB among South West residents. Median treatment delays were 35 days for presentation and 25 days for healthcare delay. Multivariable analysis identified that longer presentation delays were associated with being aged 35–50 or over 80 years old. Longer healthcare delays were associated with increasing age (hazard ratio [HR]: 0.99, 95% CI: 0.98–0.99), being employed (HR: 0.71, 95% CI: 0.52–0.97) and people currently or historically deprived of their liberty and accommodated by a prison (HR: 0.33, 95% CI: 0.20–0.54). Shorter healthcare delays were associated with sputum smear positivity (HR: 1.54, 95% CI: 1.16–2.06) and smoking (HR: 1.64, 95% CI: 1.22–2.22). Delays between symptom onset and TB treatment remain an important public health problem in the region. Factors have been identified that can be investigated to reduce presentation and healthcare delays. Detailed analyses were valuable in disaggregating key populations. Further mixed-methods research would be warranted to further understand these associations.

## Introduction

Significant progress has been made in reducing tuberculosis (TB) in England, with a decline from 15.6 incident persons with TB per 100,000 population (95% confidence interval (CI) 15.3–15.9) in 2011 to 7.3/100,000 (95% CI 7.1–7.5) in 2020 [1,2]. Post-pandemic, an increase in cases has however been observed with 8.5 incident persons with TB per 100,000 population (95% CI 8.3–8.7) reported in 2023 [3].

England is now classified as a low incidence country for TB by the World Health Organisation (WHO) [1]. Despite these improvements, disparities in the burden of TB remain. Between 2020 and 2023 rates of TB were still higher among the most deprived and those born outside the UK and over 13% of persons with TB reported each year had at least one social risk factor [1,3]. A sustained focus must be maintained on reducing TB in England, in order to decrease inequalities and meet the targets set out in the TB Action Plan for England 2021–2026 [4] and by the WHO for elimination by 2035 [5].

Timely access to treatment is essential to preventing and ending TB at a population level, and delays between symptom onset and treatment starting increase the risk of transmission and impair individual outcomes [1]. Delays can occur before individuals present to the healthcare system following onset of TB disease (presentation delay) and subsequently between initial presentation and treatment starting (healthcare delay). Healthcare delays can be separated into diagnostic delay (between presentation and TB diagnosis) and treatment delay (between TB diagnosis and treatment starting).

In England, TB delays are known to particularly affect those aged over 65 years, women, individuals residing outside London and those born in the UK [1]. Ethnicity has been found to act as an effect modifier for the association between deprivation and overall delay to starting treatment [6]. For presentation delays specifically, language barriers have been associated with longer delays [7]. Shorter healthcare delays have been associated with sputum smear positivity, residence in the UK under two years, secondary care referral and male sex in South East England [7].

The South West region is the least densely populated region in England [8], with a resident population of 5.7 million [9]. The region has less poverty and deprivation than the average in England [9] and a lower proportion of non-UK born individuals (9.4% in 2017 in comparison to 14.4% in England) [10]. TB rates declined from 6.1 (95% CI 5.4–6.8) incident persons with TB per 100,000 population in 2013 to 2.9 (95% CI 2.5–3.4) in 2020, but increased in 2023 to 3.7 (95% CI 3.2–4.2), which is more similar to the pre-COVID-19 figure [3]. Despite being a low incidence area for TB, approximately one third (34.4%) of people with pulmonary TB (pTB) in the South West had a delay of over 4 months between symptom onset and treatment starting in 2020 [11], in comparison to 32.4% in England and 25.9% in the North East (another low incidence area for TB in England) [1]. Greater than 30% of persons with pTB resident in the South West experienced a delay in excess of 4 months between 2016 and 2020 (range 34.4% in 2020–45.3% in 2017). South West England had the largest proportion of persons with TB with a delay of over 4 months in 2017 and 2018 [1]. Low incidence areas can have particular barriers to preventing and ending TB that may adversely impact delays, such as geographical barriers to accessing services in rural populations and a lack of dedicated TB service infrastructure [12].

This study aimed to identify exposures associated with presentation and healthcare delays to receiving treatment for pTB for persons notified in South West England between 2015 and 2020 in order to make targeted recommendations to prevent and end TB across the region. The objectives are to: 1) describe the total median delay, by presentation and healthcare delay; 2) describe and identify factors contributing towards the delays using univariable and multivariable analysis.

## Methods

We conducted a retrospective cohort study. All confirmed persons with pTB notified from 2015 to 2020, resident in South West England and recorded in the Enhanced Tuberculosis Surveillance (ETS) system in England were included. All persons with TB in England must legally be notified to ETS by a clinician within 72 hours of diagnosis [13,14]. Persons with TB in London are notified to the London TB register and then imported to ETS [1]. Persons with TB are notified if they are culture positive or diagnosed clinically [1,14]. ETS includes clinical and demographic data and is maintained by UKHSA (previously Public Health England). ETS commenced in 1999 as a paper-based format and an online system was introduced in 2009 [15]. Persons with drug-resistant TB were included from 2015 to 2019 due to a one-year lag before the cleaned dataset is available, as persons with drug resistant TB require a longer follow-up period. Persons with TB were excluded if they were de-notified, identified post-mortem, aged less than 15 years, had a known non-*Mycobacterium tuberculosis* species, exclusively had non-pulmonary TB or did not start treatment. Persons with TB aged less than 15 years were excluded as it is likely there are differences in their access to care, including barriers and facilitators and there are a low number of children with TB [1].

### Data cleaning

Extensive data cleaning was undertaken by the national TB team in the UKHSA. Further details are available in the UKHSA TB annual report 2021 (Appendix III) [1]. Data cleaning includes checking for inconsistencies in dates between symptoms onset, diagnosis and outcome. Duplicates are removed at multiple stages, including the involvement of regional teams, probabilistic matching of personal identifiers and linkage with lab sample dates [1]. Additional data checks were performed including examining the expected range and format of variables and identifying duplicates.

### Variables

pTB was defined as those with pTB, with or without non-pulmonary TB. Exposures of interest were identified *a priori* based on the literature review, discussions within the study team and available fields in the ETS dataset. All exposure variables were binary or categorical except for age, which was continuous. Prison, problem drug use and homelessness refer to both historical and current exposure. Smoking was classified as current smokers. Year referred to year of notification. The Index of Multiple Deprivation (IMD) categorised deprivation at a small local area level (Lower layer Super Output Areas) and had five levels ranging from the most deprived (1) to least deprived (5). Local authority included the 14 local authorities in the South West. Comorbidities included diabetes, hepatitis B, hepatitis C, chronic liver disease, chronic renal disease, immunosuppression, biological therapy and transplantation. UK-born and time in UK were combined to avoid multicollinearity. Culture and resistance were also combined as samples needed to have been cultured to obtain a resistance result. Occupation was recoded from a categorical to a binary variable, as was urban or rural residence.

Outcome variables were presentation delay and healthcare delay in days. Presentation delay was defined as the time period between symptom onset and first presentation to a healthcare service about their TB symptoms (not necessarily a TB service). Symptom onset is defined as the date symptoms were first noted by a person with TB in relation to their TB diagnosis. Symptoms would be assessed by clinicians in line with NICE guidelines [13]. First presentation date is defined as when the person with TB first visited a GP, A&E or other health professional facility in relation to their TB symptoms. Both symptoms onset and first presentation are user entered variables. Healthcare delay is defined as the time between first presentation to healthcare and TB treatment start date. Treatment start is a user entered variable, documented by the reporting clinician and is the date initial treatment was started in the UK. Delays of 0 days were recoded to a random number between 0 and 1 for the analysis, as the time of day for onset and the outcome variables was not available.

### Statistical analysis

#### Descriptive analysis.

Presentation and healthcare delays were analysed separately throughout, with the exception of the median total delay and interquartile range (IQR). Continuous variables were analysed using mean, median, interquartile ranges, minimum and maximum values and categorical data using percentages by outcome variable. The proportion of total delay attributable to presentation, diagnostic and treatment delays were calculated using the separate median delays as numerator and sum of the median delays as a denominator.

#### Missing data.

Missing data were examined using proportions and cross-tabulations for exposure and outcome variables, including cross-tabulations between the exposure variables. Odds ratios, 95% confidence intervals and p-values (Wald test for binary variables and likelihood ratio test (LRT) for categorical variables) were calculated using logistic regression to identify differences in missing data for the outcome variables between the levels of categorical variables.

#### Inferential analysis.

Cox proportional hazards regression was used for both the univariable and multivariable analysis. A hazards ratio <1 indicated a longer delay, whereas a ratio >1 indicated a shorter delay as there is a higher risk of the event (presentation or treatment). Local authority was included as a frailty term in the univariable and multivariable Cox proportional hazards regression models where possible. The magnitude and approximate LRT p-value of the term from comparing models with and without the frailty were assessed to determine its contribution to the model. The frailty term was not included in the final model if the theta value was close to 0 and the LRT of theta p-value was > 0.05. If the frailty term could not be included, a comparison between a model with or without a cluster term was included; p-values were then obtained from Wald tests.

The proportional hazards assumption was tested at both the univariable and multivariable stage. It was assessed using Kaplan-Meier survival curves and log-log plots which were plotted for presentation and healthcare delays by each exposure variable. Parallel lines indicated the proportional hazards assumption was met. Log rank tests were also used and a Chi-squared p-value of ≥0.05 indicated that the assumption was met, but it did not account for the frailty term. The proportional hazards test with the Schoenfeld residuals was also used for each exposure variable and outcome as this accounted for the frailty. A p-value of >0.05 was interpreted as the assumption being met.

Age was the only continuous exposure variable. An LRT was used to identify the suitability of the linear form of age by also including quartic, cubic or quadratic functions and assessing which term was the best fit for the model; this was conducted in the univariable analysis, at the start of the multivariable model building and in the final model.

#### Univariable analysis.

Univariable Cox proportional hazards ratios and confidence intervals were calculated for each exposure variable; using Wald p-values for binary variables and LRT p-values for categorical variables with three or more levels. Potential effect modifiers were assessed using LRT and a p-value ≤0.05 was considered a significant interaction. *A priori* interactions between sex and year and sex and UK-born/time since entry for presentation delays and ethnic group and IMD quintile for healthcare delays were examined based on the findings from previous studies in England [6,7].

#### Multivariable analysis.

Cox proportional-hazards models were fitted using maximum likelihood and forward stepwise selection. Sex and age were included in the models *a priori.* Exposure variables were added into the model in order of the smallest p-value for variables with no missing data and then the variables with missing data in order of p-value. The plausibility of the relationship between the variable and outcome was considered. The contribution of the variable to the model was assessed using LRT. Variables with an LRT p-value ≤0.05 were retained in the model. All exposures were considered as potential confounders. Variables with an LRT p-value >0.05 were assessed for confounding and included if they altered the hazard ratio of one or more parameters in the model by ≥20%. The contribution of the *a priori* interaction terms were evaluated in the final model using LRTs. If a quartic or cubic form of age was included in the final model, a plot was produced to show the effect on outcome of age. P-values for the final variables in the model were obtained using LRTs. Sensitivity analyses excluded delays between symptom onset and treatment starting over two years (730 days) and the year 2020 separately for both presentation and healthcare delays. Stata 17 [16] was used for the data cleaning, management and statistical analysis.

### Ethical statement

Ethical approval was not required for this study as an analysis of routine surveillance data to inform public health action by UKHSA. Data were accessed for data analysis purposes from 20/01/2022. The lead author had access to identifiable information as part of their role working for the UKHSA. UKHSA staff can access confidential patient information without consent for the purpose of recognizing trends and monitoring and managing infectious diseases under section 251 of the National Health Service Act 2006 and Regulation 3 of the associated Health Service (Control of Patient Information) Regulations 2002.

## Results

### Total persons with TB and delay

**Fig 1:**
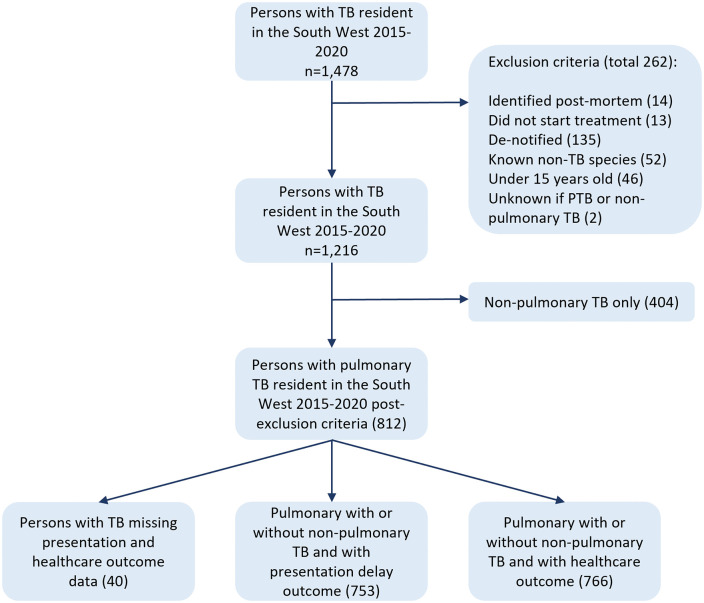
Flow chart outlining the number of persons with pTB in the South West between 2015-2020 included in the analysis.

Between 2015 and 2020 there were 1,478 persons with TB in South West residents. After applying the exclusion criteria there were 1,216 cases of TB, of which 66.8% (812/1,216) were persons with pTB. Of the 812 persons with pTB, 60.5% (491/812) were male and the median age was 43 (IQR: 30–60). There were 753 persons with pTB with data on presentation delay and 766 with healthcare delay (Fig 1). Table 1 outlines the characteristics of the persons with pTB.

**Table 1:** Characteristics of the persons with pTB in the South West included in the study, 2015-2020.

Exposure	Level	Presentation outcome data available (n = 753)		Healthcare outcome data available (n = 766)		Presentation and healthcare outcome data available (n = 747)	
		n	%	n	%	n	%
Age (median, IQR in years)	–	42	30 - 60	42	30-60	42	30-60
Sex	Female	300	39.8	306	40.0	298	39.9
	Male	453	60.2	460	60.1	449	60.1
Rurality	Urban	655	87.0	668	87.2	654	87.6
	Rural	98	13.0	98	12.8	93	12.5
IMD quintile	1	288	38.3	294	38.4	288	38.6
	2	132	17.5	139	18.2	132	17.7
	3	31	4.1	32	4.2	30	4.0
	4	129	17.1	129	16.8	127	17.0
	5	173	23.0	172	22.5	170	22.8
Employed	No	305	40.5	309	40.3	302	40.4
	Yes	421	55.9	430	56.1	418	56.0
	Missing	27	3.6	27	3.5	27	3.6
Ethnic group	White	453	60.2	460	60.1	448	60.0
	Black Caribbean	12	1.6	12	1.6	12	1.6
	Black African	93	12.4	93	12.1	93	12.5
	Black other	7	0.9	7	0.9	7	0.9
	Indian	62	8.2	62	8.1	62	8.3
	Pakistani	20	2.7	21	2.7	20	2.7
	Bangladeshi	12	1.6	13	1.7	12	1.6
	Chinese	12	1.6	12	1.6	12	1.6
	Mixed/ other	77	10.2	80	10.4	76	10.2
	Missing	5	0.7	6	0.8	5	0.7
UK born/non – UK born and time since entry (years)	UK born	364	48.3	373	48.7	362	48.5
	0–1	67	8.9	69	9.0	65	8.7
	2–5	80	10.6	81	10.6	80	10.7
	6–10	87	11.6	86	11.2	86	11.5
	11+	110	14.6	110	14.4	110	14.7
	Missing	45	6.0	47	6.1	44	5.9
Year	2015	166	22.1	169	22.1	166	22.2
	2016	136	18.1	137	17.9	135	18.1
	2017	121	16.1	123	16.1	120	16.1
	2018	106	14.1	110	14.4	106	14.2
	2019	137	18.2	140	18.3	136	18.2
	2020	87	11.6	87	11.4	84	11.2
Prison	No	623	82.7	633	82.6	620	83.0
	Yes	47	6.2	47	6.1	47	6.3
	Missing	83	11.0	86	11.2	80	10.7
Homeless	No	641	85.1	653	85.3	639	85.5
	Yes	56	7.4	56	7.3	54	7.2
	Missing	56	7.4	57	7.4	54	7.2
Drug use	No	637	84.6	649	84.7	634	84.9
	Yes	57	7.6	58	7.6	57	7.6
	Missing	59	7.8	59	7.7	56	7.5
Alcohol misuse	No	667	88.6	676	88.3	664	88.9
	Yes	34	4.5	38	5.0	34	4.6
	Missing	52	6.9	52	6.8	49	6.6
Comorbidity	No	628	83.4	638	83.3	623	83.4
	Yes	125	16.6	128	16.7	124	16.6
Smoker	No	402	53.4	411	53.7	400	53.6
	Yes	193	25.6	196	25.6	191	25.6
	Missing	158	21.0	159	20.8	156	20.9
Previous diagnosis of TB	No	670	89.0	684	89.3	666	89.2
	Yes	56	7.4	56	7.3	56	7.5
	Missing	27	3.6	26	3.4	25	3.4
Sputum smear	Negative	172	22.8	178	23.2	172	23.0
	Positive	245	32.5	246	32.1	242	32.4
	Missing	336	44.6	342	44.7	333	44.6
Culture & resistance	Not culture confirmed	216	28.7	224	29.2	216	28.9
	Culture confirmed no resistance	413	54.8	417	54.4	409	54.8
	Culture confirmed & resistant	45	6.0	46	6.0	45	6.0
	Culture confirmed, resistance unknown	79	10.5	79	10.3	77	10.3

The total median delay was 91.5 days (IQR: 45–179.5). As a percentage of the total delay, 64.5% was attributable to the presentation delay, 34.4% to the diagnostic delay and 1.1% to the treatment starting delay.

### Presentation delay

Of the 753 people with data for presentation delay, the median delay was 35 days (IQR: 10–95 days). A Kaplan-Meier plot for presentation delay is available in S1 Fig. Median presentation delays by exposure variable are presented in Table 2.

**Table 2:** Median (and IQR) presentation and healthcare delays, univariable hazards ratios, 95% confidence intervals and p-values for persons with pTB in the South West of England from 2015 to 2020 for each exposure variable.

Exposure	Level	Presentation delay (n = 753^a^)						Healthcare delay (n = 766^a^)					
		n	Median delay in days	IQR^b^	Hazard ratio^c^	95% CI	p-value^d^	n	Median delay in days	IQR^b^	Hazard ratio	95% CI	p-value^d^
Age	–	–	–	–	0.998	0.994–1.002	0.4	–	–		0.99	0.99–1.00	0.005
Age squared	–	–	–	–	1.0002	1.0000–1.0004	0.02	–	–		–	–	–
Age cubed	–	–	–	–	0.99998	0.99997–0.999995	0.004	–	–		–	–	–
Sex	Female	300	36	11–99	Ref	–	0.60	306	28	8–80	Ref	–	0.13
	Male	453	33	10–91	1.04	0.90–1.21		460	23	7–68	1.13	0.96–1.33	
Rurality	Urban	655	35	10–92	Ref	–	0.23	668	24	7–69	Ref	–	0.51
	Rural	98	35.5	15–109	0.87	0.69–1.09		98	30.5	11–83	0.94	0.78–1.13	
IMD quintile	1	288	35	10–111	Ref	–	0.94	294	25.5	7–68	Ref	–	0.68
	2	132	31	14–95	1.10	0.89–1.35		139	29	9–74	0.92	0.76–1.13	
	3	31	34	14–103	1.15	0.79–1.67		32	16.5	5.5–53	1.15	0.80–1.66	
	4	129	33	4–86	1.17	0.95–1.44		129	28	9–84	0.90	0.73–1.11	
	5	173	39	14–91	1.05	0.87–1.27		172	21.5	7–68	0.94	0.78–1.14	
Employed	No	305	35	10–103	Ref	–	0.88	309	28	9–72	Ref	–	0.38
	Yes	421	34	11–95	1.01	0.87–1.17		430	24	7–69	1.07	0.92–1.24	
	Missing	27	39	14–86	–	–		27	11	3–60	–	–	
Ethnic group	White	453	36	10–109	Ref	–	0.67	460	25	7–74	Ref	–	0.91
	Black Caribbean	12	67.5	31–131	0.78	0.44–1.38		12	18.5	7–77	1.10	1.01–1.20	
	Black African	93	30	11–63	1.09	0.87–1.36		93	21	6–65	1.19	0.98–1.43	
	Black other	7	79	4–118	1.00	0.47–2.11		7	29	10–52	1.32	0.76–2.30	
	Indian	62	31	8–99	1.04	0.80–1.36		62	33	15–67	0.95	0.80–1.13	
	Pakistani	20	38.5	6–79	0.80	0.51–1.26		21	32	16–47	1.00	0.75–1.33	
	Bangladeshi	12	26.5	11–65	1.38	0.78–2.45		13	32	9–51	1.12	0.74–1.70	
	Chinese	12	40.5	30–67	1.08	0.61–1.91		12	17	7–73	1.0	0.47–2.11	
	Mixed/ other	77	30	14–64	1.17	0.92–1.48		80	27	8–68	1.10	0.90–1.34	
	Missing	5	30	12–96	–	–		6	6.5	2–13	–	–	
UK born/non – UK born and time since entry years	UK born	364	37	12–111	Ref		0.11	373	30	9–82	Ref	–	0.04
	0–1	67	22	3–55	1.41	0.08–1.84		69	18	5–62	1.39	1.07–1.80	
	2–5	80	35.5	14–123	0.94	0.74–1.20		81	24	7–59	1.14	0.90–1.46	
	6–10	87	35	10–81	1.09	0.86–1.38		86	26	9–56	1.35	1.06–1.71	
	11+	110	37	14–96	1.04	0.84–1.28		110	22.5	6–84	1.09	0.88–1.36	
	Missing	45	30	14–60	–	–		47	16	6–41	–	–	
Year	2015	166	34	15–92	Ref	–	0.24	169	17	6–54	Ref	–	<0.001
	2016	136	39	13–107	0.93	0.74–1.16		137	23	7–67	0.83	0.67–1.05	
	2017	121	35	9–126	0.86	0.68–1.09		123	27	9–67	0.74	0.58–0.93	
	2018	106	39	10–87	1.04	0.81–1.32		110	49.5	8–102	0.59	0.46–0.75	
	2019	137	28	8–52	1.17	0.93–1.46		140	32	8–108	0.62	0.49–0.78	
	2020	87	37	10–109	0.96	0.74–1.25		87	25	9–55	0.86	0.66–1.11	
Prison	No	623	35	10–96	Ref	–	0.21	633	24	7–72	Ref	–	0.08
	Yes	47	49	26–110	0.83	0.61–1.11		47	38	10–78	0.81	0.64–1.02	
	Missing	83	29	4–79	–	–		86	29	8–66	–	–	
Homeless	No	641	35	13–99	Ref	–	0.92	653	27	8–74	Ref	–	0.28
	Yes	56	38.5	8–101	1.01	0.77–1.34		56	15.5	5–40	1.16	0.88–1.53	
	Missing	56	29	4–81	–	–		57	27	7–65	–	–	
Drug use	No	637	34	10–94	Ref		0.06	649	27	8–73	Ref	–	0.30
	Yes	57	70	28–120	0.77	0.58–1.01		58	20	7–52	1.15	0.88–1.51	
	Missing	59	28	5–90	–	–		59	16	6–65	–	–	
Alcohol misuse	No	667	35	11–93	Ref	–	0.24	676	27	8–73	Ref	–	0.50
	Yes	34	47.5	19–137	0.81	0.57–1.15		38	15.5	4–37	1.12	0.80–1.57	
	Missing	52	22.5	5–91	–	–		52	16	5–54	–	–	
Comorbidity	No	628	35	11–99	Ref	–	0.29	638	25	7–72	Ref	–	0.40
	Yes	125	34	10–79	1.11	0.91–1.35		128	25	11–58	1.09	0.90–1.31	
Smoker	No	402	35.5	10–96	Ref	–	0.51	411	31	10–79	Ref	–	0.05
	Yes	193	37	15–110	0.94	0.79–1.12		196	21	7–67	1.15	1.00–1.33	
	Missing	158	30	7–87	–	–		159	14	5–54	–	–	
Previous diagnosis of TB	No	670	35.5	13–96	Ref	–	0.18	684	26	7–70	Ref	–	0.54
	Yes	56	21.5	6–90	1.21	0.92–1.59		56	22.5	8–75	1.09	0.83–1.43	
	Missing	27	28	4–82	–	–		26	16	6–38	–	–	
Sputum smear	Negative	–	–		–	–	–	178	33.5	9–77	Ref	–	0.003
	Positive	–	–		–	–	–	246	14	5–46	1.34	1.10–1.63	
	Missing							342	33.5	10–80	–	–	
Culture & resistance	Not culture confirmed	–	–		–	–	–	224	36	9–85	Ref	–	0.20
	Culture confirmed no resistance	–	–		–	–	–	417	22	7–66	1.16	0.99–1.37	
	Culture confirmed & resistant	–	–		–	–	–	46	26.5	8–64	1.27	0.92–1.74	
	Culture confirmed, resistance unknown				–	–	–	79	21	9–55	1.21	0.94–1.57	

^a^A frailty term was included for local authority as a random effect for each of the univariable hazards ratios, or a cluster term if the frailty could not be included.

^b^Age rounded to 3 decimal places.

^c^Rounded to 0 decimal places.

^d^Excluding the missing values.

**Fig 2:**
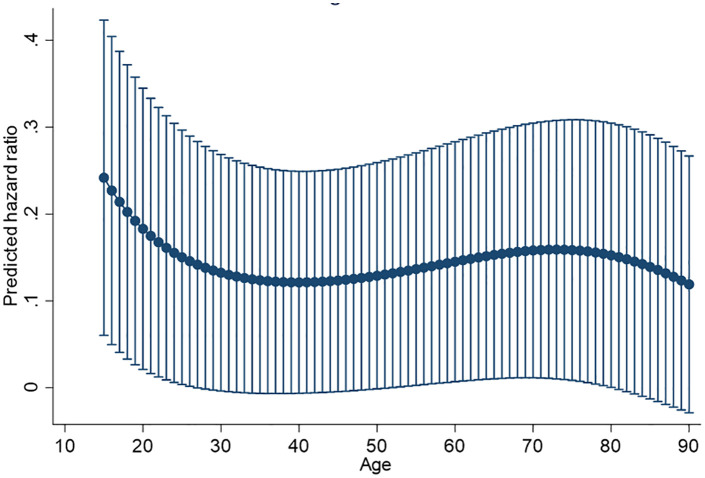
The relationship between age cubed and presentation delay for treatment of pTB in South West England, 2015-2020 adjusted for sex. Note: lower predicted hazards ratios indicated longer delays between symptom onset and presentation to healthcare. Hazards ratios are lower for those aged 35-50 years and over 80 years old.

All exposure variables met the proportional hazards assumption in the univariable analysis, except for drug use. Univariable analysis identified that age was the only variable associated with presentation delays; a cubic function was the most appropriate (hazards ratio (HR): 1.00 for age, age squared and age cubed) (Table 2). Multivariable analysis confirmed that a cubic function of age was the only variable associated with presentation delays after adjusting for sex (Table 3). Fig 2 illustrates the relationship between age and presentation delays. Longer presentation delays were associated with being between approximately aged 35–50 years and being aged over 80 years old, as the predicted hazards ratios were lower in this age range. The association in the multivariable analysis was similar to that found in the univariable analysis. Interactions between sex and year, or sex and UK-born/time since entry did not add to the final model and so were not included. The proportional hazards assumption was valid for the final multivariable model. Local authority was not included as a frailty term in the final model as it did not contribute to the model. Sensitivity analyses excluding the year 2020 and those with a delay over two years did not alter the findings in the final model; the association of age with presentation delays remained cubic and sex was not associated.

**Table 3:** Final adjusted models with hazards ratios for i) presentation delay ii) healthcare delay by exposure for persons with pTB in the South West of England from 2015–2020.

i) Model for presentation delay (n = 753)				
Exposure	Level	Adjusted hazards ratio	95% CI	LRT p-value
Sex	Male	1.03	0.89–1.19	0.69
Age	–	0.88	0.82–0.94	0.003^a^
Age squared	–	1.00	1.00	
Age cubed	–	1.00	1.00–1.00	
**ii) Model for healthcare delay (n = 301)**				
**Exposure**	**Level**	**Adjusted hazards ratio**	**95% CI**	**LRT p-value**
Sex	Male	1.05	0.79–1.38	0.75
Age	–	0.99	0.98–0.99	0.001
Year	2015	Ref	–	0.06
	2016	1.20	0.77–1.89	
	2017	1.05	0.68–1.65	
	2018	0.70	0.43–1.13	
	2019	0.71	0.45–1.11	
	2020	1.03	0.64–1.66	
Sputum smear	Negative	Ref	–	–
	Positive	1.54	1.16–2.06	0.003
Prison	No	Ref	–	–
	Yes	0.33	0.20–0.54	<0.001
Smoker	No	Ref	–	–
	Yes	1.64	1.22–2.22	0.001
Employed	No	Ref	–	–
	Yes	0.71	0.52–0.97	0.03
Comorbidity	No	Ref	–	–
	Yes	1.37	0.92–2.02	0.12
**Ethnic group**				
White	IMD 1	Ref	–	–
	IMD 2	1.35	0.91–2.00	<0.001
	IMD 3	1.13	0.49–2.59	
	IMD 4	1.48	0.85–2.59	
	IMD 5	1.21	0.78–1.88	
Black – Caribbean	IMD 1	0.71	0.27–1.84	
	IMD 2	0.96	0.32–2.86	
	IMD 3	0.80	0.21–3.00	
	IMD 4	1.05	0.33–3.38	
	IMD 5	0.85	0.28–2.59	
Black – African	IMD 1	1.27	0.71–2.27	
	IMD 2	1.75	0.60–5.14	
	IMD 3	1.43	0.49–4.17	
	IMD 4	8.41	1.10–64.59	
	IMD 5	2.06	0.62–6.91	
Black – Other	IMD 1	4.33	0.56–33.22	
	IMD 2	5.84	0.71–47.83	
	IMD 3	4.88	0.53–44.85	
	IMD 4	6.42	0.74–55.70	
	IMD 5	5.23	0.64–42.98	
Indian	IMD 1	1.95	0.96–3.98	
	IMD 2	2.29	0.80–6.54	
	IMD 3	1.74	0.23–13.43	
	IMD 4	1.00	0.23–4.24	
	IMD 5	1.35	0.41–4.45	
Pakistani	IMD 1	1.27	0.58–2.77	
	IMD 2	1.71	0.68–4.35	
	IMD 3	1.43	0.44–4.65	
	IMD 4	1.88	0.67–5.32	
	IMD 5	1.48	0.34–6.54	
Bangladeshi	IMD 1	1.05	0.41–2.73	
	IMD 2	0.93	0.21–4.16	
	IMD 3	1.20	0.32–4.44	
	IMD 4	0.69	0.09–5.30	
	IMD 5	1.27	0.42–3.87	
Chinese	IMD 1	2.70	0.80–9.05	
	IMD 2	3.64	0.95–13.96	
	IMD 3	3.05	0.70–13.30	
	IMD 4	0.30	0.04–2.34	
	IMD 5	35.29	4.27–291.51	
Mixed/other	IMD 1	1.67	0.89–3.13	
	IMD 2	0.53	0.25–1.13	
	IMD 3	14.53	1.75–120.48	
	IMD 4	3.46	1.50–8.00	
	IMD 5	4.36	1.67–11.35	

^a^p-value for improvement of fit over the quadratic function.

Ref: Reference group.

There was no evidence to suggest the other variables were associated with presentation delays, including sex, year, and UK-born/non-UK born status.

### Healthcare delay

Of the 766 people with data for healthcare delay, the median healthcare delay was 25 days (IQR 7–70 days). A Kaplan-Meier plot for presentation delay is available in S2 Fig. Median healthcare delays by exposure variable are presented in Table 2.

All exposure variables met the proportional hazards assumption in the univariable analysis, except for homeless and culture/resistance. Univariable analysis identified that longer healthcare delays were associated with the person with TB being reported in 2017 (HR: 0.74 (95% CI: 0.58–0.93), 2018 (HR: 0.59 (95% CI: 0.46–0.75) and 2019 (HR: 0.62 (95% CI: 0.49–0.78) (p = 0.0001) in comparison to 2015 (Table 2). Shorter delays were associated with those who were sputum smear positive (HR: 1.34, 95% CI: 1.10–1.63, p = 0.003) or non-UK born and had recently arrived in the past year in the UK (HR: 1.39 (95% CI: 1.07–1.80) or who had lived in the UK for 6−10 years (HR: 1.35 (95% CI: 1.06–1.71) (p = 0.04). A linear function of age could be used in this model as higher order polynomials did not fit significantly better using the process described in the methods.

In the multivariable analysis, after adjusting for sex, sputum smear positive persons with TB remained associated with shorter delays (HR: 1.54, 95% CI: 1.16–2.06) as did being a smoker (HR: 1.64, 95% CI: 1.22–2.22) (Table 3). Longer healthcare delays were associated with people currently or previously deprived of their liberty and accommodated by a prison (HR: 0.33, 95% CI: 0.20–0.54), being employed (HR: 0.71, 95% CI: 0.52–0.97) and older age (HR: 0.99, 95% CI: 0.98–0.99). Year and UK-born status/time since entry to the UK were no longer associated. Ethnic group was identified as an effect modifier for deprivation, but there were very small numbers in the sub-groups. The proportional hazards assumption was valid for the final multivariable model. There was no evidence to suggest the other variables were associated with healthcare delays including sex, culture and resistance and comorbidity.

Local authority was not included as a random effect in the final model as the variation was adequately explained by the fixed effects. Excluding the year 2020 in the sensitivity analysis meant being employed was no longer associated with a longer healthcare delay. Excluding delays over two years had no impact on the associations identified in the multivariable analysis.

### Missing data and a delay of zero days

Of the 812 persons with pTB, 3.9% (32/812) were missing total delay, 7.3% (59/812) presentation delay and 5.7% (46/812) healthcare delay. Neither presentation delay or healthcare delay were missing completely at random, as there were associations between the missing data and alcohol misuse (p = 0.002) and employment (p = 0.02) for presentation delays and rural residence (p = 0.004), employment (p = 0.002) and IMD (p = 0.01) for healthcare delays (Table 4). Smoking was introduced as a variable in ETS in mid-2015, but any variation was not significant for presentation or healthcare delay. Delays were recoded from zero to a random number between zero and one for 13.5% (102/753) records for presentation delay and 1.4% (11/766) records for healthcare delay.

**Table 4:** A comparison between persons with pTB in the South West missing presentation and healthcare delay by exposure variable, 2015-2020.

Exposure	Level	Missing presentation delay					Missing healthcare delay				
		N	%	OR	95% CI	p-value	N	%	OR	95% CI	p-value
Total cases	All	59	7.27	–		–	46	5.67	–	–	–
Sex	Female	21	6.54	1.20	0.69–2.08	0.52	15	4.67	1.37	0.73–2.59	0.32
	Male	38	7.74				31	6.31			
Rural	No	46	6.56	1.89	0.98–3.62	0.06	33	4.71	2.69	1.37–5.28	0.004
	Yes	13	11.71				13	11.71			
Prison	No	42	6.32	*	–	–	32	4.81	*	–	–
	Yes	0	0				0	0			
Homeless	No	46	6.70	1.24	0.48–3.26	0.66	34	4.95	1.71	0.65–4.56	0.28
	Yes	5	8.20				5	8.20			
Drug use	No	48	7.01	0.23	0.03–1.72	0.15	36	5.26	*	–	–
	Yes	1	1.72				0	0			
Alcohol misuse	No	42	5.92	3.74	1.63–8.58	0.002	33	4.65	2.16	0.73–6.40	0.17
	Yes	8	19.05				4	9.52			
Comorbidity	No	51	7.51	0.79	0.37–1.70	0.54	41	6.04	0.61	0.24–1.57	0.30
	Yes	8	6.02				5	3.76			
Smoker	No	29	6.73	0.79	0.39–1.61	0.52	20	4.64	0.84	0.36–1.94	0.68
	Yes	11	5.39				8	3.92			
Previous diagnosis	No	47	6.56	1.27	0.49–3.33	0.62	33	4.60	1.85	0.70–4.93	0.22
	Yes	5	8.20				5	8.20			
Year	2015	12	6.74	Ref	–	0.97	9	5.06	Ref	–	0.09
	2016	14	9.33	1.42	0.64–3.18		13	8.67	1.78	0.74–4.29	
	2017	9	6.92	1.03	0.43–2.52		7	5.38	1.07	0.39–2.95	
	2018	6	5.36	0.78	0.29–2.15		2	1.79	0.34	0.07–1.61	
	2019	9	6.16	0.91	0.37–2.22		6	4.11	0.80	0.28–2.32	
	2020	9	9.38	1.43	0.58–3.53		9	9.38	1.94	0.74–5.07	
UK born/non – UK born and time since entry (years)	UK born	35	8.77	–	–	0.09	26	6.52	–	–	0.57
	0–1	5	6.94	0.78	0.29–2.05		3	4.17	0.62	0.18–2.12	
	2–5	3	3.61	0.39	0.12–1.30		2	2.41	0.35	0.08–1.52	
	6–10	4	4.40	0.48	0.17–1.38		5	5.49	0.83	0.31–2.23	
	11+	7	5.98	0.66	0.29–1.53		7	5.98	0.91	0.39–2.16	
Employment	Unemployed	33	9.76	0.50	0.29–0.88	0.02	29	8.58	0.35	0.18–0.67	0.002
	Employed	23	5.18				14	3.15			
IMD quintile	1	18	5.88	–	–	0.17	12	3.92	Ref	–	0.01
	2	11	7.69	1.33	0.61–2.90		4	2.80	0.71	0.22–2.23	
	3	3	8.82	1.55	0.43–5.55		2	5.88	1.53	0.33–7.15	
	4	7	5.15	0.87	0.35–2.13		7	5.15	1.33	0.51–3.45	
	5	20	10.36	1.85	0.95–3.59		21	10.88	2.99	1.44–6.23	
Ethnic group	White	37	7.55	–	–	0.65	30	6.12	Ref	–	0.72**
	Black Caribbean	1	7.69	1.02	0.13–8.06		1	7.69	1.28	0.16–10.16	
	Black African	3	3.13	0.39	0.12–1.31		3	3.13	0.49	0.15–1.65	
	Black other	0	0	–	–		0	0	–	–	
	Indian	2	3.13	0.39	0.09–1.68		2	3.13	0.49	0.12–2.12	
	Pakistani	3	13.04	1.84	0.52–6.47		2	8.70	1.46	0.33–6.52	
	Bangladeshi	2	14.29	2.04	0.44–9.46		1	7.14	1.18	0.15–9.32	
	Chinese	2	14.29	2.04	0.44–9.46		2	14.29	2.56	0.55–11.94	
	Mixed/other	7	8.33	1.11	0.48–2.59		4	4.76	0.77	0.26–2.24	
Sputum smear	Negative	–	–	–	–	–	8	4.30	1.63	0.69–3.83	0.26
	Positive	–	–	–	–	–	18	6.82			
Culture & resistance	Not culture confirmed	–	–	–	–	–	14	5.88	Ref	–	0.73
	Culture confirmed no resistance	–	–	–	–	–	22	5.01	0.84	0.42–1.68	
	Culture confirmed & resistant	–	–	–	–	–	3	6.12	1.04	0.29–3.78	
	Culture confirmed, resistance unknown	–	–	–	–	–	7	8.14	1.42	0.56–3.64	

*Omitted due to null values (one level of variable with no missing data for the outcome).

**Black other ethnic group excluded as no missing data for healthcare delay.

## Discussion

### Delays between symptom onset and treatment start

This study found that while significant progress has been made towards reducing the rate of TB in South West England, delays between symptom onset and treatment starting remain an important public health issue for the region. The total median delay of 91.5 days for individuals aged 15 years or over with pTB in the South West in 2020 was 12.5 days longer than the delay for England in 2020 [1] and longer than historic delays [6]. This delay of almost three months has consequences for transmission risk and morbidity.

Disaggregating the total delay by presentation and healthcare delay assists in designing targeted interventions. Average presentation delay in the South West was longer than the healthcare delay which has been found nationally [3] and elsewhere in England [17] but not universally [18]. In comparison to another low incidence area in England between 2011 and 2015, the average presentation delay in the South West was five days longer, while the average healthcare delay was 15 days shorter [7]. There was a wide range of delays and this was also seen, albeit to a lesser extent, in South East England [7]. The median healthcare delay in the South West was still 25 days and so there remains significant opportunities for improvement. Unfamiliarity with TB disease and a low index of clinical suspicion in low incidence settings could contribute to delays in confirming the clinical diagnosis and prompt referral to TB services.

### Factors contributing towards the delays

Presentation delays are likely to reflect barriers to accessing healthcare. Barriers include individual, organisational and structural factors, for example, a perceived lack of risk of infection, stigma, physical access to healthcare and challenging contexts for the individuals [19].

Older age has previously been linked to longer presentation [7,17] and overall delays in starting treatment in England [6]. In the South West, presentation delays for persons with pTB in those aged 15 years and over were associated with being over 80 years of age, but also being aged 35–50 years old. The reasons for longer delays in these age ranges requires further investigation. Presentation delays could be reduced in older people by raising awareness of TB symptoms in the health and social care sectors, to improve recognition of the symptoms among those caring for older individuals, for example in care homes. Age is frequently included as a categorical variable in studies; this finding highlights the importance of including age as a continuous variable and testing the linear relationship with the outcome in order to elucidate more complex relationships and avoid drawing incorrect conclusions. Longer presentation delays have previously been associated with other factors, including a history of being deprived of their liberty and accommodated by a prison, which the authors suggested could be due to a chaotic lifestyle or poor past experience with healthcare professionals [17]; we did not identify this in our study.

Healthcare delays could be reduced by strategies within the healthcare setting targeting those who are older, employed, non-smokers and who are currently, or were previously deprived of their liberty and accommodated by a prison. Smoking was associated with a shorter healthcare delay; smokers may have more comorbidities and clinicians could have greater concerns about lung disease leading to accelerated investigations and referrals. In line with previous findings [7], smear positivity was associated with a shorter healthcare delay, providing reassurance that persons with the most infectious pTB should have shorter delays. Barriers could however remain to prompt treatment for those who are smear negative and could still transmit TB. Being deprived of their liberty and accommodated by a prison was associated with healthcare delays and further analyses would be warranted to examine whether delays affect people currently, or previously, deprived of their liberty and accommodated by a prison, or both. To note only a small proportion of those with healthcare outcome data were deprived of their liberty and accommodated by a prison (6.1%). Our findings concurred with previous studies indicating that older age is associated with a longer healthcare delay, potentially due to increased comorbidities complicating the diagnosis [17].

Being employed was associated with a longer healthcare delay. The South West had higher levels of employment than other regions in England during the time period under investigation [9]. Individuals who are unemployed could be a very specific population where there may be a higher suspicion of TB. It is notable that this association only existed when the year 2020 was included in the analysis, when COVID-19 restrictions were frequently in place in England. Individuals employed during this time could have been key workers, fearful of losing their job or reluctant to access healthcare during this period and so may not have attended follow-up appointments. Significant disruption to TB services during the COVID-19 pandemic have been reported globally leading reduced diagnosis of persons with TB in 2020 [20–23]. The difference in the impact of the COVID-19 between and within countries has been recognised by other authors [22] and it is useful to observe particular associations with employment in South West England to further this understanding. Further research to understand this association further would be useful.

Ethnicity was an effect modifier for the association between IMD and healthcare delays, but this finding needs to be treated with caution due to the small numbers in the sub-groups once persons with TB are disaggregated by ethnicity and IMD. Findings for specific ethnic groups could also be explained by clusters, which may be more likely to occur among individuals of a similar ethnic group. Contact tracing is recommended for people who are a close contacts of someone with pTB or laryngeal TB [24], which may mean that persons with TB identified are in the same ethnic group (for example, family contacts). Persons with TB identified as part of a cluster may access TB treatment more quickly due to public health action, as there will be a higher index of suspicion of TB among contacts of known persons with TB.

It was perhaps surprising that additional exposure variables were not associated with delays, particularly presentation delay and some findings differ from previous studies in London [17], nationally [25] and South East England [7]. Geographical and temporal differences are likely to be important, as TB epidemiology [1], population characteristics and client case-mix are different in London and the TB rate in England has declined since previous study periods.

### Strengths and limitations of the study

Strengths of our study include the use of Cox proportional hazards regression, as time-to-event data accounts for the time leading up to an event and has greater statistical power than logistic regression using a binary outcome. This may explain some of the differing results to studies which used binary outcomes with arbitrary cut-offs. Cox regression was used over accelerated failure time models which require more assumptions. ETS is a comprehensive, representative and high-quality dataset and all known persons with TB in the South West during the time period should have been included. Sensitivity analyses were included to determine whether there were any impacts of the extreme outliers with long delays or the COVID-19 pandemic in 2020. Social risk factors were disaggregated in this study; imprisonment has been combined with other social risk factors in previous studies and so individual associations may have been missed.

Limitations of the study include the analysis being restricted to the variables available in ETS, including for example more structural and system level factors, although the dataset does contain a wide range of variables from enhanced surveillance. The exclusion of the small proportion of individuals with pTB without an outcome could cause a bias. Certain variables had a large proportion of missing data, namely prison, smoking and sputum smear status for those with healthcare delays, which could introduce a bias. Further investigation into the reasons for the missing data would be warranted. The final model included several of these variables, resulting in fewer observations being included. Exposure variables which were missing not at random for the delays could also have introduced a bias and the analysis therefore doesn’t conform to the Missing at Random (MAR) or Completely Missing at Random (MCAR) assumptions. Other limitations included the symptom onset date being self-reported, which could be subject to recall bias and the lack of drug-resistant persons with TB in the dataset for 2020, due to the year lag before the data are available. A total of 13 individuals did not initiate treatment and were excluded, which could introduce a bias. Some of the persons with TB may be part of a cluster, which could result in shorter delays. A cluster variable could not be included as the completeness of the variable in ETS was low. The study was based in the South West and so may not be applicable to other areas in England.

### Further research

Conducting a regional analysis and identifying specific barriers to target to reduce delays was important as TB service provision is configured regionally. Further discussion of these findings with regional TB leads will assist in identifying individual, organisational or structural issues that contribute to the presentation or healthcare delays. Mixed-method or qualitative studies would be particularly useful in understanding the reasons for the delays identified and the range observed in delays and could be used to investigate the reasons for those who didn’t start treatment. Further analyses would be useful to examine the factors associated with delays between symptom onset and treatment start for non-pulmonary TB and children aged under 15 years. Any potential association between the severity of illness due to TB and coinfection with HIV would also be useful to investigate. The methods used in this study, including age as a continuous variable, could be applied to other regions in England to see if similar associations exist.

## Conclusions

Substantial progress has been made in reducing the incidence of TB in England and the South West is a low incidence area. Despite this, delays between symptom onset and treatment start remain an important public health issue and could jeopardise progress towards the WHO End TB strategy [5], particularly with the observed increase in the incident number of persons with TB in the South West and England in 2023 [3]. Our study identified factors associated with presentation and healthcare delays, that require further investigation. Discussions with regional TB teams and further applied research could assist with understanding the reasons behind these delays and designing appropriate interventions. Replicating this study and the detailed analyses undertaken could assist other low incidence areas in England and internationally to identify exposures to investigate to reduce delays between symptom onset and treatment start.

## Supporting information

S1 Fig Kaplan-Meier plot demonstrating the time to presentation by proportion of participants who have not presented. Note: x axis presents delays up until 286 days (95% of delays).
(TIF)

S2 Fig Kaplan-Meier plot demonstrating the time to treatment by proportion of participants who have not received treatment. Note: x axis presents delays up until 240 days (95% of delays).
(TIF)
