# Supplementary figures and images for: Exposures associated with tuberculosis presentation and healthcare delays in South West England, 2015–2020
Source: PLoS One. 2026 Jan 7;21(1):e0340187. doi: 10.1371/journal.pone.0340187 (PMC12779073; doi:10.1371/journal.pone.0340187)

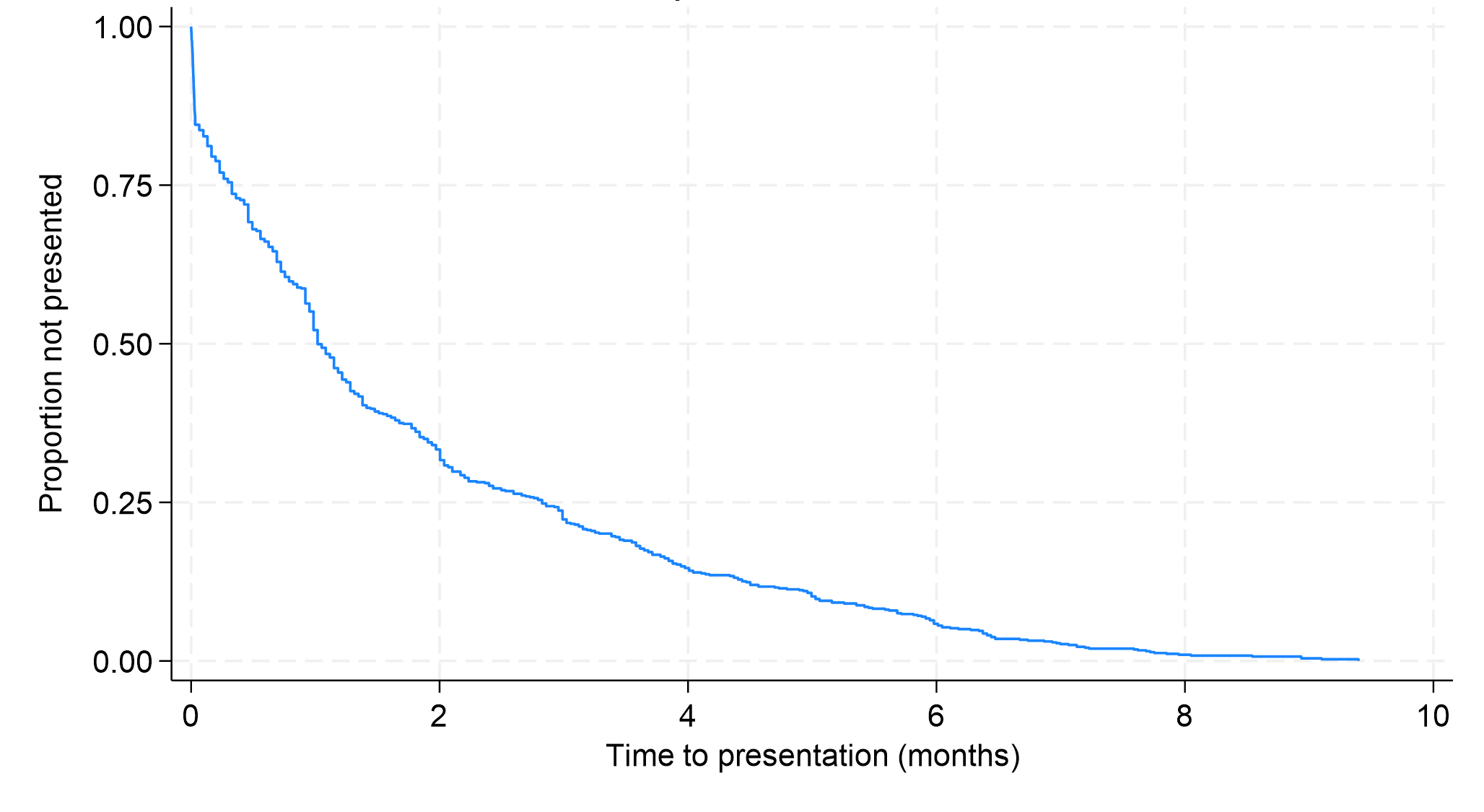

Supplement: S1 Fig — Note: x axis presents delays up until 286 days (95% of delays). (TIF) [file pone.0340187.s001.tif]

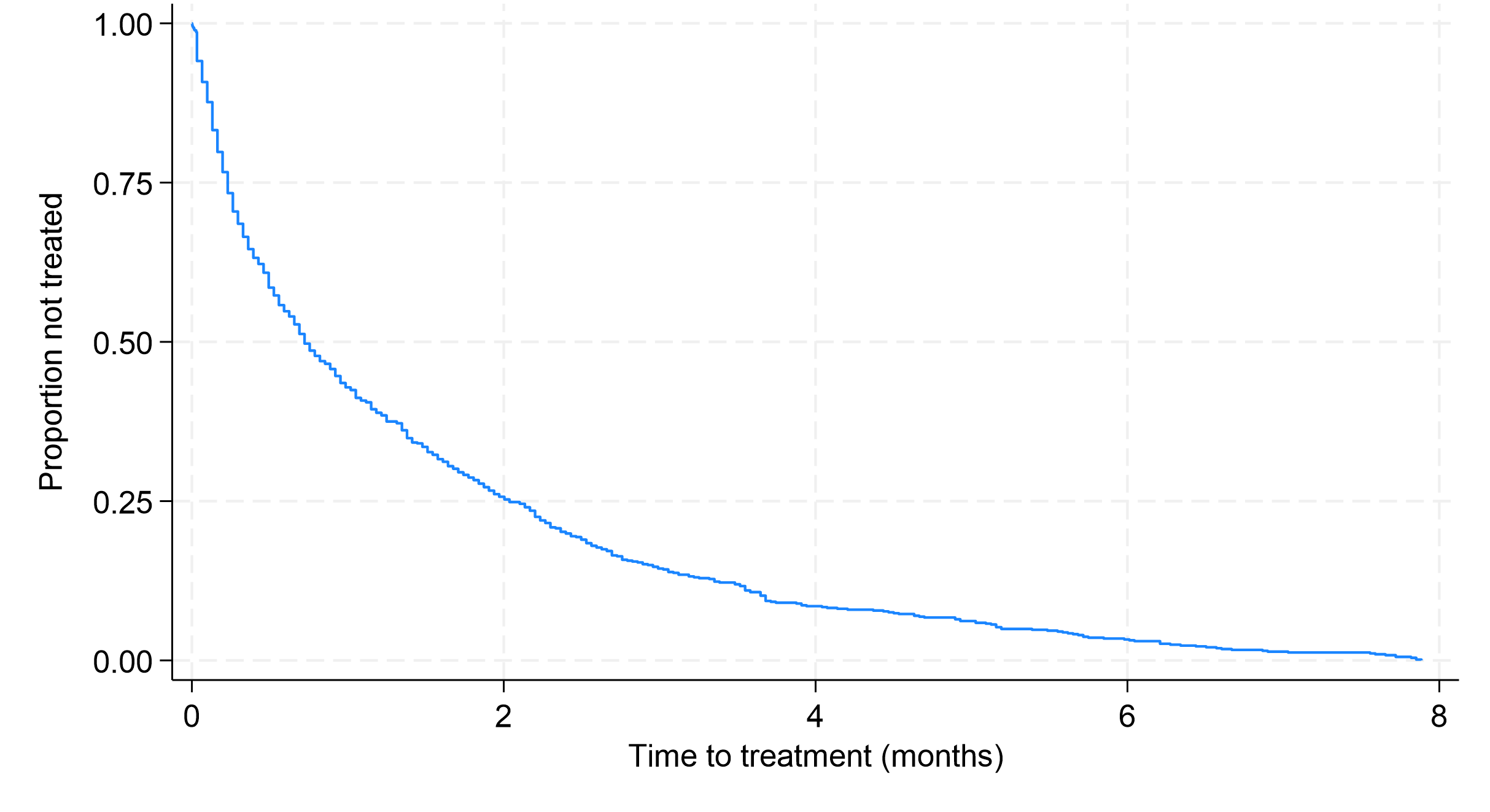

Supplement: S2 Fig — Note: x axis presents delays up until 240 days (95% of delays). (TIF) [file pone.0340187.s002.tif]
